# Quercetin induces pathogen resistance through the increase of salicylic acid biosynthesis in *Arabidopsis*

**DOI:** 10.1080/15592324.2023.2270835

**Published:** 2023-10-30

**Authors:** Jonguk An, Sun Ho Kim, Sunghwa Bahk, Minh Le Anh Pham, Jaemin Park, Zakiyah Ramadany, Jeongwoo Lee, Jong Chan Hong, Woo Sik Chung

**Affiliations:** Division of Applied Life Science (BK21 Four Program), Plant Molecular Biology and Biotechnology Research Center, Gyeongsang National University, Jinju, Republic of Korea

**Keywords:** Flavonoid, NPR1, Pathogen resistance, Quercetin, Salicylic acid

## Abstract

Quercetin is a flavonol belonging to the flavonoid group of polyphenols. Quercetin is reported to have a variety of biological functions, including antioxidant, pigment, auxin transport inhibitor and root nodulation factor. Additionally, quercetin is known to be involved in bacterial pathogen resistance in *Arabidopsis* through the transcriptional increase of *pathogenesis-related* (*PR*) genes. However, the molecular mechanisms underlying how quercetin promotes pathogen resistance remain elusive. In this study, we showed that the transcriptional increases of *PR* genes were achieved by the monomerization and nuclear translocation of nonexpressor of pathogenesis-related proteins 1 (NPR1). Interestingly, salicylic acid (SA) was approximately 2-fold accumulated by the treatment with quercetin. Furthermore, we showed that the increase of SA biosynthesis by quercetin was induced by the transcriptional increases of typical SA biosynthesis-related genes. In conclusion, this study strongly suggests that quercetin induces bacterial pathogen resistance through the increase of SA biosynthesis in *Arabidopsis*.

## Introduction

Flavonoids are widely distributed polyphenolic secondary metabolites with multiple biological activities in plants.^1^ Flavonoids are associated with the development and defense of plants, including the colors and aromas of flowers, pollen fertility, auxin transport inhibitors, UV filters, allelopathy, insect resistance and antioxidants.^1–7^ Flavonoids, such as naringenin, kaempferol and quercetin, are produced in the early stages of the flavonoid biosynthesis pathway and appear to be widely present in *Arabidopsis*.^8–10^ Recent studies have reported that flavonoids such as naringenin, kaempferol, quercetin and rutin induce resistance to pathogens through the SA signaling pathway^11–14^. Notably, quercetin has also been found to participate in the defense response against pathogens such as *Pseudomonas syringae* and *Pyricularia oryzae*.^14,15^ Nevertheless, the molecular mechanisms to explain how quercetin induces pathogen resistance remain unclear.

Salicylic acid (SA) is a crucial defense hormone that plays a pivotal role in the immune response and systemic acquired resistance.^16–18^ The pathogens trigger the activation of SA production, which stimulates immune response. SA is produced by two distinct pathways, the isochorismate and the phenylalanine ammonialyase pathways.^17^ The key transcription factors, *SARD1* and *CBP60g*, positively regulate the SA biosynthesis genes, including *ICS1* and *EDS5*.^17,18^ SA promotes *EDS1* and *PAD4* expression, which initiates a positive feedback loop that further amplifies SA accumulation.^19,20^

NPR1 functions as a major regulator in the SA signaling pathway.^16,17,21^ NPR1 is involved in the transcriptional activations of *PR* genes that are the representative marker genes of the disease resistance against biotrophic pathogens in plants.^22,23^ NPR1 is mainly located in the cytoplasm as an oligomer form in the absence of SA. However, NPR1 is monomerized by the increase of SA level in response to the pathogen infection.^24,25^ The monomeric NPR1 is then translocated into nucleus from cytoplasm, where it interacts with TGA transcription factors that lead to the transcriptional activation of *PR* genes.^17,24,26^

In our study, we discovered that quercetin triggers the up-regulation of *PR* genes by the monomerization and nuclear translocation of NPR1. Additionally, we observed that the nuclear translocation of NPR1 is achieved by the accumulation of SA. Conclusively, we suggested that quercetin induces pathogen resistance by the activation of the SA-dependent signaling pathway through the increase of SA biosynthesis.

## Materials and methods

### Plant materials and growth conditions

The following *Arabidopsis* lines were used in the study: Columbia-0 (Col-0) and *35S:NPR1-GFP* in *npr1–2*. The seeds were washed with 70% EtOH for 1 min, followed by 10 min in 1/10-diluted commercial bleach (0.4% NaOCl) and five washes with sterile distilled water. The seedlings were grown on Murashige-Skoog (MS) salts and vitamins^27^, 2.0% sucrose and 0.8% agar. The plates were incubated for 3 d at 4°C in the dark and then grown in a growth chamber (16 h light/8 h dark cycle, light intensity of ~120 μmol m^−2^ s^−1^) at 22°C for 14 d.

### Bacterial growth curve assays

*Pseudomonas syringae* pv. *tomato* DC3000 was used in this study. For observation of disease symptoms, four-week-old plant leaves were pre-infiltrated with 0.5 µM flg22 or 100 µM quercetin using a 1 mL needless syringe. After two days, mock or pre-infiltrated plants were inoculated with bacterial suspension of 5 × 10^5^ colony-forming units CFU/mL in 10 mM MgCl_2_ using a 1 mL needless syringe. After three days, leaf discs (total area 0.5 cm^2^) were collected by cork borer. The samples were ground in 10 mM MgCl_2_ and plated in serial dilution on Pseudomonas Agar F (MB cell, Korea). A two-tailed Student’s t-test was used for statistical analysis.

### Protein extraction and immunoblot analysis

Immunoblot analysis was performed as described.^28^ Total protein was extracted with lysis buffer (50 mM HEPES, pH 7.5, 5 mM EGTA, 5 mM EDTA, 25 mM NaF, 50 mM-glycerophosphate, 1 mM Na_3_VO_4_, 2 mM DTT, 2 mM PMSF, 5% glycerol, 1% Triton X-100 and protease inhibitor). After centrifugation at 12,000 g for 15 min at 4°C, total protein concentrations were measured by Bradford assay (Bio-Rad, USA). To detect NPR1, proteins (30 µg) were fractionated in 8% gel by SDS-PAGE and electrophoretically transferred to a PVDF membrane. The anti-NPR1 antibodies (1:5,000; Abiocode, USA) were used as primary antibodies and horseradish peroxidase-conjugated anti-rabbit antibodies as secondary antibodies (1:10,000). Signal detection was visualized using an ECL kit (Bio-Rad, USA).

### Total RNA extraction and qPCR analysis

For transcription level analysis, Col-0 plants were treated with 0.5 µM flg22 or 100 µM quercetin for 24 h. All samples were immediately placed in liquid nitrogen and stored at −80°C. Total RNA was extracted using an RNA purification kit (Macherey-Nagel, Germany). cDNA was synthesized from 5 μg total RNA using a RevertAid Reverse Transcriptase (Thermo Scientific, USA). The expressions of *PR1*, *PR2*, *ICS1*, *EDS1*, *PAL1* and *CBP60g* were quantified by qPCR (CFX384 Real-Time System, Bio-Rad, USA) as previously described^29^. The tubulin was used as an internal control. The primers used for qPCR are shown in Supplementary Table S1.

### Fluorescence microscopy

The fluorescence imaging of leaves was generated using the confocal laser-scanning microscope (Olympus FV1000). Plants were treated with 200 μM SA or 100 μM quercetin for 3 h. 488 nm laser line was used to detect GFP. GFP fluorescence was taken through images from 500 to 539 nm at a gain setting of 556.

### Measurement of salicylic acid

The quantification of SA levels was accomplished through high-performance liquid chromatography (HPLC) employing a reverse-phase C18 Gemini column for subsequent HPLC – ESI–MS/MS analysis, utilizing an API 4000 instrument (SCIEX). This methodology closely followed a previously established protocol.^30^ About 50 mg of powdered, frozen sample was extracted with 500 μl of extraction solvent 2-propanol/H_2_O/concentrated HCl (2:1:0.002, v/v/v) containing d6-SA, an internal standard for SA, respectively 24 h at 4°C. Dichloromethane (1 mL) was added to the supernatant and then centrifuged at 13,000 g for 5 min at 4°C. The lower phase was dried under nitrogen and resolved in pure methanol. The fully dissolved extract was transferred to a liquid chromatography vial with a reduced volume, following thorough vortexing and sonication.

### Statistical analysis

Statistical analysis in this study was conducted using Prism 9 (GraphPad, USA). Each experiment was carried out in triplicate with three sets of biological samples. One-way analysis of variation (ANOVA) with Tukey’s post hoc test was performed for multi-sample experiments with one variable. Two-way ANOVA with Tukey’s post hoc test was performed for multi-variable analyses. All results are expressed as mean ± SD.

## Results

### The physiological level of quercetin is an inducer of bacterial pathogen resistance

**Figure 1.**
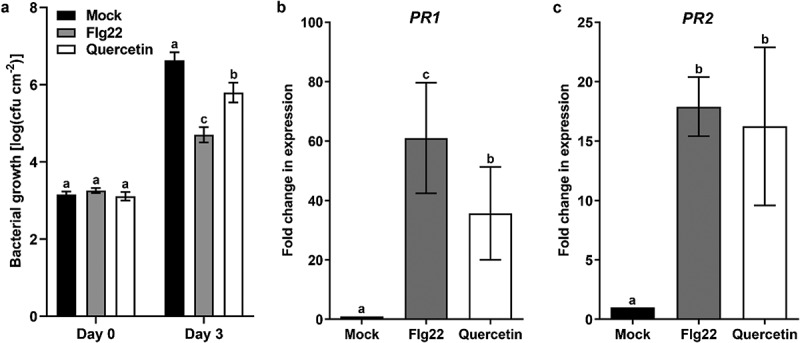
Quercetin induces an effective pathogen resistance against *Pst* DC3000. (a) the bacterial growth in Col-0 plants. Two days before the infiltration of *Pst* DC3000, four-week-old plants were infiltrated with 0.5 µM flg22 or 100 µM quercetin. Bars represent mean values (SD) of colony-forming units (CFU) per square centimeter from biological replicate samples derived from different plants. Different letters indicate significant differences (*P* < .001) tested by two-way ANOVA with Tukey’s post hoc test. (b, c) qPCR analysis of *PR1* and *PR2* transcription levels in Col-0 plant treated with 0.5 µM flg22 or 100 µM quercetin for 24 h. The relative gene expression level was normalized to that of the tubulin internal control. Error bars indicate the SD of three biological replicates. Different letters indicate significant differences (*P* < .05) tested by one-way ANOVA with Tukey’s post hoc test.

It has been reported that bacterial pathogen resistance is induced by flavonoids such as rutin, naringenin, kaempferol and quercetin.^11–14^ To test pathogen resistance, excessive concentrations of quercetin are used in the previous report.^14^ The concentration of quercetin can be increased up to approximately 100 µM by pathogen infection or laser irradiation.^31,32^ To test whether the physiological level of quercetin can induce bacterial pathogen resistance, we examined the pathogen resistance against *Pseudomonas syringae* pv. tomato DC3000 (*Pst* DC3000) after treatment with 100 µM quercetin. As a result, we found that the proliferation of *Pst* DC3000 was inhibited by the pre-treatment of quercetin (Figure 1a). This result suggests that quercetin induces pathogens resistance at the physiological concentration. To test whether quercetin-mediated pathogen resistance at the physiological level of concentrations is related to the expression of *PR* genes, we analyzed the transcript levels of *PR* genes after treatment with 100 µM quercetin. Interestingly, the gene expressions of *PR1* and *PR2* were 35.6-fold and 16.2-fold up-regulated by the treatment with quercetin, respectively (Figure 1b, c). Taken together, these results suggest that quercetin induces pathogen resistance by transcriptional up-regulation of *PR* genes at the physiological level of concentrations.

### The monomerization and nuclear translocation of NPR1 is induced by quercetin

**Figure 2.**
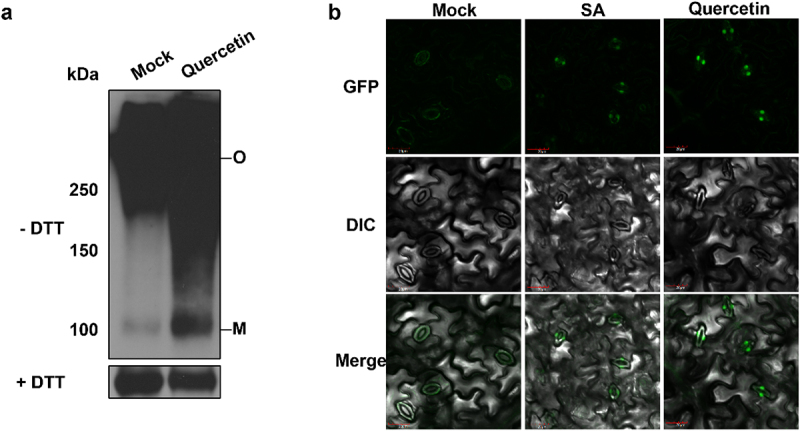
NPR1 is monomerized and translocated to the nucleus by quercetin. (a) NPR1 is monomerized by quercetin. Total proteins were extracted from two-week-old seedlings of *35S:NPR1-GFP/npr1–2* treated with 100 μM quercetin for 3 h and detected using anti-NPR1 antibodies. SDS-PAGE was performed with or without DTT in the sample buffer. (b) NPR1 is translocated to the nucleus by quercetin. *35S:NPR1-GFP/npr1–2* were treated with 200 μM SA or 100 μM quercetin for 3 h. The fluorescence was observed by a confocal microscope. Scale bar = 20 μm. The experiment was repeated three times with similar results.

The activation of NPR1 is due to the conformational change from oligomeric form to monomeric form, which is induced by the direct binding to SA.^24,33^ The monomerization of NPR1 is involved in the transcriptional up-regulation of *PR* genes.^21^ To investigate whether quercetin induces the monomerization of NPR1, we examined the level of NPR1 monomer by immunoblot. As previously reported, the oligomeric form of NPR1 is highly detected before the treatment with quercetin. However, the monomeric form of NPR1 is accumulated by the treatment with quercetin (Figure 2a). This result suggests that NPR1 is monomerized by quercetin.

The nuclear translocation of NPR1 after the monomerization is required for the induction of *PR* genes.^24,34^ Therefore, we examined whether quercetin increases the nuclear translocation of NPR1 by confocal microscopy. As a result, the fluorescence of NPR1-GFP was mostly visible in the cytoplasm of guard cells before the treatment with quercetin, whereas the fluorescence was highly detected in the nucleus after the treatment with quercetin (Figure 2b). This result showed that NPR1 is translocated from the cytoplasm to the nucleus by quercetin. Taken together, we conclude that quercetin triggers the transcriptions of *PR* genes through the monomerization and nuclear translocation of NPR1.

### Quercetin induces the biosynthesis of SA by increasing the transcription level of SA biosynthesis-related genes

**Figure 3.**
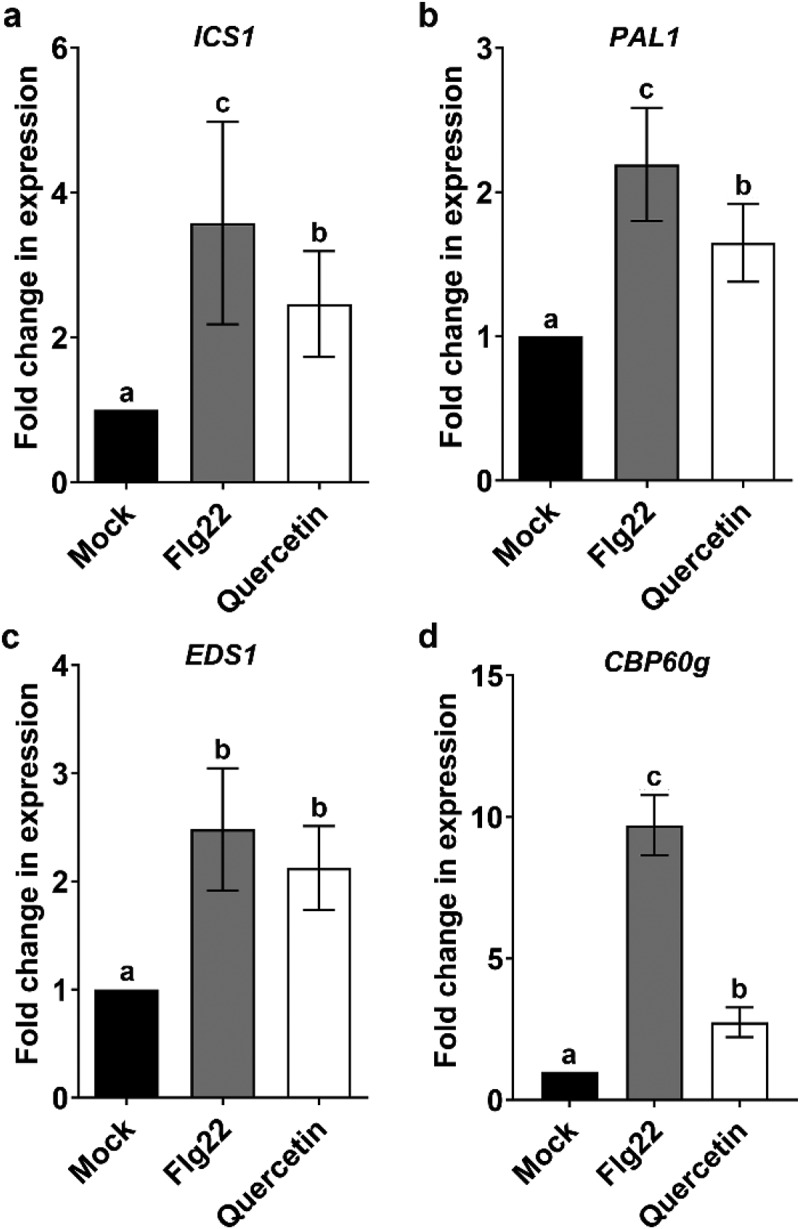
SA biosynthesis-related genes are up-regulated by quercetin. qPCR analysis *ICS1*, *PAL1*, *EDS1* and *CBP60g* transcription levels in Col-0 plant treated with 0.5 µM flg22 or 100 µM quercetin for 24 h. The relative gene expression level was normalized to that of the tubulin internal control. Error bars represent the SD of three biological replicates. Different letters indicate significant differences (*P* <.05) tested by one-way ANOVA with Tukey’s post hoc test.

**Figure 4.**
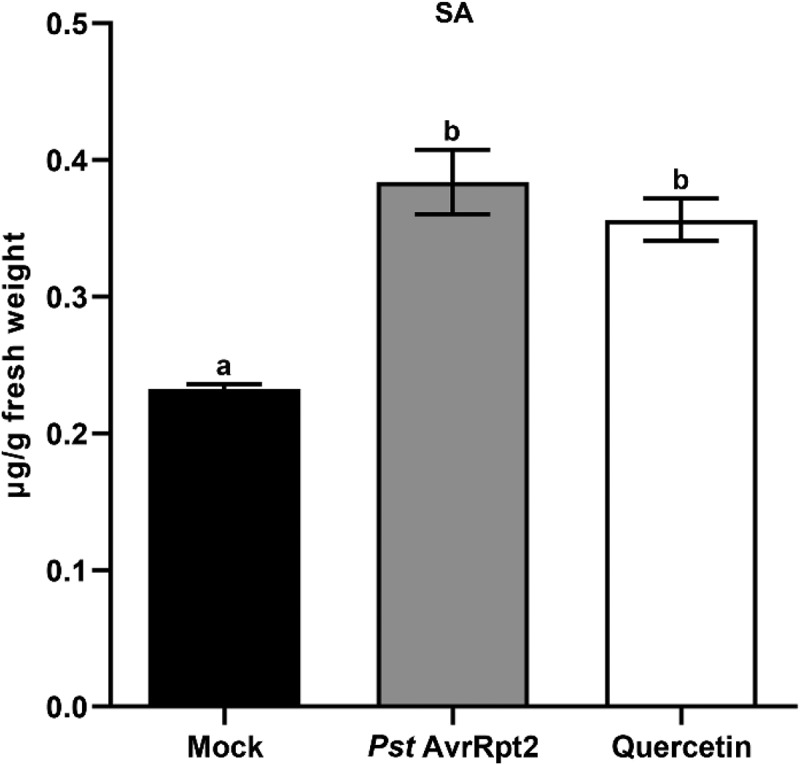
The accumulation of SA is highly induced by quercetin. The level of SA in *Pst* DC3000-inoculated or 100 μM quercetin-treated Col-0 leaves at 24 hpi (hour post-infection) using HPLC-MS/MS. Error bars indicate the SD of three biological replicates. Different letters indicate significant differences (*P* <.001) tested by one-way ANOVA with Tukey’s post hoc test.

It was well established that bacterial pathogen resistance can be achieved by the accumulation of SA.^16^ Flavonoids are known to promote pathogen resistance through the SA-dependent signaling pathway.^11–13^ Therefore, we hypothesized that quercetin up-regulates the biosynthesis of SA. To test this hypothesis, we first examined the transcription levels of SA biosynthesis-related genes after the treatment with quercetin by qRT-PCR. As a result, we found that four SA biosynthesis-related genes, *ICS1, PAL1, EDS1* and *CBP60g*, are about 2.5-fold, 1.6-fold, 2.1-fold and 2.7-fold up-regulated by quercetin, respectively (Figure 3). This result strongly indicates that quercetin induces SA biosynthesis through the induction of SA biosynthesis-related genes. Therefore, we measured the level of SA by HPLC-MS/MS after the treatment with quercetin. As a result, we found that the level of SA is about 2-fold increased by quercetin (Figure 4). The increased level of SA by quercetin is similar to the level in the case of treatment with an avirulence pathogen, the *Pst* DC3000 avrRpt2. This result suggests that quercetin induces the biosynthesis of SA by the transcriptional increase of SA biosynthesis-related genes.

## Discussion

### Quercetin can induce resistance to pathogens at low concentrations

Flavonoids are major secondary metabolites found in many fruits, vegetables, seeds, grains tea and wine.^1,8^ Several flavonoids such as naringenin, kaempferol, quercetin, rutin and riboflavin have been reported to induce the resistance against various pathogens in *Arabidopsis*.^11–14,35^ However, in the previous study, the excess concentration of quercetin was applied to examine the bacterial pathogen resistance.^14^ In this study, we showed that the pathogen resistance was obtained by the treatment with the physiological concentration of quercetin (Figure 1). Previous reports have shown that the physiological level of naringenin and kaempferol induced pathogen resistance against bacteria.^11,12^ This result suggests that the physiological concentration of quercetin is sufficient to induce pathogen resistance. The biological functions of several different flavonoids in pathogen resistance have examined at only a standalone level.^11–14,35,36^ The synergistic effects of the different combinations of flavonoids, or the combinations of flavonoids with other defense compounds would be studied. Furthermore, it should be studied how flavonoid is recognized and transduced for disease resistance.

### Quercetin induces resistance to pathogens through the accumulation of SA

SA is known to be accumulated by pathogen infection and stimulates plant immunity to the bacterial pathogen in local and systemic tissues.^37,38^ Previously, quercetin was able to activate pathogen resistance through ROS accumulation and induction of *PAL1* gene.^14^ In this study, we showed that quercetin increased the accumulation of SA by the transcriptional increase of SA biosynthesis-related genes (Figures 3 and 4). Similar results reported that SA biosynthesis was induced by naringenin, kaempferol, riboflavin and thiamine.^11,12,35,39^ These results suggest that flavonoid-induced pathogen resistance is achieved by the accumulation of SA. Previously, it has been reported that pathogen resistance was induced by the accumulations of flavonoids in poplar, tomato and mango.^40–43^ These results implicate that pathogen resistance can be commonly induced through the accumulation of SA in response to flavonoids in plants. Therefore, more detail experiments should be performed in different plants in near future.

### Quercetin confers pathogen resistance through the activation of NPR1

The activation of NPR1 is known to be an essential process of SA-mediated immune responses.^38,44^ Since quercetin could not induce the pathogen resistance in *npr1*mutant, it was suggested that NPR1 is involved in quercetin-induced disease resistance.^14^ However, the molecular mechanism to explain how quercetin activates NPR1 is not elucidated. In this study, we showed that quercetin activates NPR1 by the monomerization and nuclear translocation (Figure 2). Similarly, naringenin and kaempferol also induce the activation of NPR1.^11,12^ Meanwhile, because several kinases, such as PKS5 and SnRK2.8, have been associated with the phosphorylation of NPR1,^21,45,46^ quercetin can be involved in the activations of these kinases. Therefore, the activities of these kinases by the treatment with quercetin should be tested in the future.

**Figure 5.**
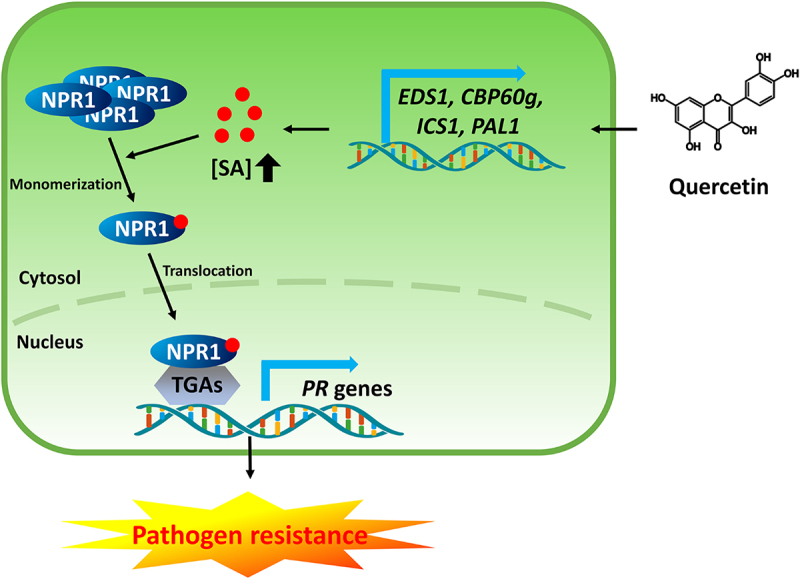
The working model explains how quercetin induces pathogen resistance. Quercetin increases the biosynthesis of SA through the transcriptional increase of SA biosynthesis-related genes. The accumulation of SA by quercetin triggers the monomerization and nuclear translocation of NPR1, resulting in the induction of *PR* genes and pathogen resistance.

Based on this study, we proposed a working model to explain how quercetin induces pathogen resistance (Figure 5). In this model, we provide evidence that quercetin induces resistance to bacterial pathogens by the accumulation of SA. The accumulated SA by quercetin induces the expression of *PR* genes through the activation of NPR1. Taken together, we conclude that quercetin induces pathogen resistance through an SA-dependent signaling pathway in *Arabidopsis*. These findings implicated that quercetin or similar flavonoids can be used to improve crop yield by increasing plant resistance against bacterial pathogens as natural compounds.

## Supplementary Material

Supplemental Material
