# Supplementary material for: Quercetin induces pathogen resistance through the increase of salicylic acid biosynthesis in Arabidopsis
Source: Plant Signal Behav. 2023 Oct 30;18(1):2270835. doi: 10.1080/15592324.2023.2270835 (PMC10761074; doi:10.1080/15592324.2023.2270835)
Supplement: Supplemental Material [file KPSB_A_2270835_SM5079.docx]

**Supplementary Table 1.** Primers used for qPCR.

| **Construct** | **Position** | **Sequence** |
| --- | --- | --- |
| **PR1** | **F** | 5'- GTGGGTTAGCGAGAAGGCTA -3' |
|  | **R** | 5'- ACTTTGGCACATCCGAGTCT -3' |
| **PR2** | **F** | 5'- CGGTACATCAACGTTGGAA -3' |
|  | **R** | 5'- GCGTAGTCTAGATGGATGTT -3' |
| **ICS1** | **F** | 5'- AGTGAATTTGCAGTCAGTCGGGAT -3' |
|  | **R** | 5'- AATCGCCTGTAGAGATGTTGT -3' |
| **EDS1** | **F** | 5'- TCGAAGGGGACATAGATTGG -3' |
|  | **R** | 5'- CTTTTCATGTACGGCCCTGT -3' |
| **PAL1** | **F** | 5'- TGTAGCGCAACGTACC -3' |
|  | **R** | 5'- GTTCGGGATAGCCGATG -3' |
| **CBP60g** | **F** | 5’-GAGCTCGTAACTTAACATTCAAGAAA-3’ |
|  | **R** | 5’-CTTATCATGTTCATCTGAATCAT-3’ |
| **Tubulin** | **F** | 5'- CCAACAACGTGAAATCGACAG -3' |
|  | **R** | 5'- TCTTGGTATTGCTGGTACTCT -3' |
